# Impact of AI Literacy on Well-Being Among Nursing Students—Mediating Roles of Empowerment and Anxiety: Cross-Sectional Study

**DOI:** 10.2196/79789

**Published:** 2025-12-22

**Authors:** Amira Alshowkan, Emad Shdaifat

**Affiliations:** 1 Community Nursing Department, College of Nursing, Imam Abdulrahman Bin Faisal University, King Faisal Street, Dammam, 1982, Saudi Arabia, 966 544312234

**Keywords:** artificial intelligence, anxiety, sex factors, well-being, structural equation modeling

## Abstract

**Background:**

The integration of artificial intelligence (AI) in health care is changing nursing practice, and it calls for the acquisition of AI literacy by students, which includes knowledge, skills, and attitudes. An understanding of the effect of AI literacy on the well-being and empowerment of students is crucial in guiding effective educational strategies.

**Objective:**

This study aims to investigate the impact of AI literacy on well-being, with psychological empowerment and anxiety serving as mediating variables. Using partial least squares structural equation modeling (PLS-SEM), this study examines gender differences within these relationships.

**Methods:**

A cross-sectional design was used, and data were gathered from 497 nursing students from Imam Abdulrahman Bin Faisal University, Saudi Arabia, via a structured online questionnaire assessing AI literacy, psychological empowerment, anxiety, and well-being. PLS-SEM was used to evaluate both the measurement and structural models, encompassing mediation and multigroup analyses based on gender.

**Results:**

The constructs demonstrated substantial reliability and validity, and the model’s fit was deemed satisfactory. Well-being was moderately accounted for (*R*²=0.41), whereas empowerment and anxiety exhibited lower levels of explained variance. All hypotheses were supported, indicating that AI literacy positively influenced empowerment and negatively affected both anxiety and well-being. Furthermore, empowerment was found to negatively impact both anxiety and well-being. The mediation effects were significant, and no gender differences were observed.

**Conclusions:**

The study demonstrates that AI literacy significantly influences psychological empowerment, anxiety, and overall well-being through both direct and indirect pathways. The findings elucidate the intricate relationships among these variables and provide evidence for the applicability of the model across genders. This underscores the critical importance of promoting AI literacy and empowerment as a means to improve well-being outcomes.

## Introduction

### Background

The rapid integration of artificial intelligence (AI) into health care is transforming both clinical practice and nursing education. As future frontline health care providers, nursing students are increasingly exposed to AI technologies that promise to enhance clinical decision-making, operational efficiency, and patient outcomes [1,2]. However, this advancement also introduces educational and psychological challenges, including the need for sufficient AI literacy, psychological empowerment, and strategies to manage emotional responses to AI integration.

Understanding how AI literacy influences psychological empowerment, anxiety, and overall well-being is essential for equipping nursing students with the competencies required in evolving health care environments [3,4]. AI literacy—defined as the knowledge, skills, and ethical understanding necessary to effectively use AI tools—is key to enabling students to engage with these technologies confidently [5,6]. Psychological empowerment, reflecting a sense of control, competence, and meaning in one’s role, may mediate the relationship between AI literacy and psychological outcomes [7].

In this study, anxiety refers to general anxiety symptoms, measured using the Generalized Anxiety Disorder 7-item (GAD-7) scale, rather than AI-specific or job-related anxiety. Although not directly tied to AI, general anxiety captures broader emotional distress, which may be influenced by students’ perceptions and experiences with AI [8]. Conversely, AI-related anxiety, defined as apprehension or fear specifically related to the use or implications of AI, has also been shown to negatively affect academic performance and mental health [9,10].

Given these dynamics, exploring the interplay among AI literacy, psychological empowerment, and general anxiety is critical for developing targeted educational and psychological interventions to support nursing students’ resilience and well-being in the era of AI.

### Literature Review

AI literacy in nursing education encompasses ethical awareness, technical knowledge, and practical skills in working with AI-driven health care technology [3,5]. The literature suggests that the readiness of nursing students in accepting AI is a function of their attitude toward technology, perceived digital literacy, and ethics [4,11]. Limited access to AI resources and knowledge gaps remain significant barriers to full integration, stemming from students’ confidence and intention to apply AI tools in practice [5,12].

Psychological empowerment is essential to enhance motivation and engagement in technology adoption. Zhang et al [7] demonstrated that perceived affordances of AI applications significantly support psychological empowerment, which leads to the adoption of AI-based medical consultations. In nursing practice, AI literacy empowerment can reduce feelings of helplessness and enhance self-efficacy, allowing for more effective learning and clinical performance [11].

Despite the potential benefits, AI adoption can evoke anxiety, characterized by uncertainty and fear about job security, capability, and moral concerns [3,10]. AI anxiety was found to be linked to poorer mental health status, increased stress, and opposition to using AI technologies [9]. In nursing students, this anxiety can undermine academic performance and decision-making, underscoring the need for targeted psychological interventions [13,14].

Readiness for AI is positively correlated with students’ well-being, as adequate preparation reduces uncertainty and improves resilience [13,15]. AI literacy with psychological empowerment enables nursing students to use AI for academic success and mental health benefits, such as burnout decrease and self-management enhancement [16,17].

This study is guided by the Job Demands-Resources (JD-R) model and Psychological Empowerment theory. The JD-R model clarifies that job demands, including AI complexity and ethical concerns, lead to strain and anxiety, whereas resources, including AI literacy and empowerment, reduce stress and increase well-being [11]. Psychological empowerment theory concentrates on 4 cognitions—meaning, competence, self-determination, and impact—which are enhanced by AI literacy and influence affective responses to technology [7]. Together, these models explain how AI literacy is a resource that enhances empowerment, reduces AI anxiety, and ultimately improves well-being in nursing students.

The significance of this study is in addressing crucial gaps in the knowledge of the psychological impact of AI adoption in nursing education. By examining the interplay among AI literacy, psychological empowerment, anxiety, and well-being, this study will provide nursing educators and policymakers with insights into effective strategies to enhance AI readiness while safeguarding students’ mental health. Empowerment and AI literacy can reduce anxiety, improve academic performance, and ready future nurses to apply AI technologies in patient care with confidence. Additionally, results can guide the development of AI-driven personalized interventions, such as mental health services and ethics education, to guarantee a skilled and resilient nursing workforce for the digital age [3,16,18].

### Research Objectives

This study seeks to explore how literacy in AI influences well-being, considering psychological empowerment and anxiety as mediating factors. Using partial least squares structural equation modeling (PLS-SEM), this study also examines potential gender differences within these relationships.

### Hypotheses

**Figure 1.**
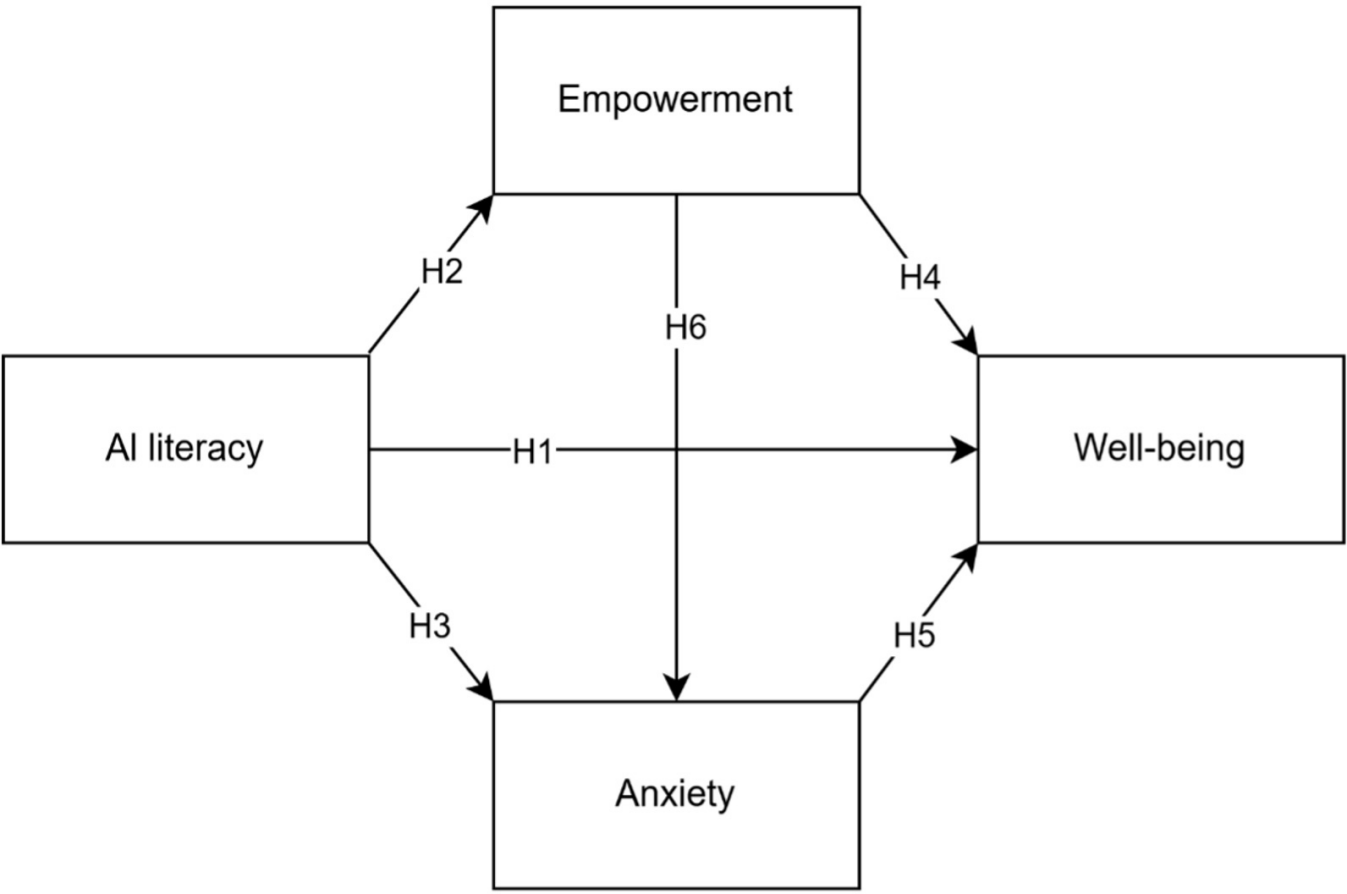
Conceptual framework and hypotheses.

The conceptual framework and study hypotheses are summarized in Figure 1.

Our study hypotheses are as follows:

- **H1:** AI literacy directly affects well-being.
- **H2:** AI literacy directly affects empowerment.
- **H3:** AI literacy directly affects anxiety.
- **H4:** Empowerment directly affects well-being.
- **H5:** Anxiety directly affects well-being.
- **H6:** Empowerment directly affects anxiety.

## Methods

### Site and Setting

This study took place among nursing students at Imam Abdulrahman Bin Faisal University in the Eastern Province of Saudi Arabia from January to April 2025.

### Sampling Size and Technique

This study concentrated on students enrolled in the College of Nursing, encompassing nursing students from the first to the fifth year, as well as those participating in their internship year. Data collection was conducted using a convenience sampling method. The sample size was determined using the Raosoft calculator [19] based on a total population of 1200 nursing students, a 95% confidence level, and a 5% margin of error. A sample of 292 nursing students was established as being representative of the population.

### Variables and Tools

This study evaluated 4 critical variables—AI literacy, psychological empowerment, anxiety, and well-being—each assessed using validated measurement instruments as detailed in the subsequent sections.

AI literacy was assessed using the Artificial Intelligence Literacy Scale (AILS) [20], which evaluates participants’ comprehension, skills, and attitudes regarding AI technologies. The scale encompasses 12 items related to AI concepts, applications, and ethical considerations. Responses were measured on a Likert-type scale, ranging from 1=strongly disagree to 5=strongly agree. Elevated scores denote a higher level of AI literacy. The Arabic version of the AILS successfully replicated the original four-factor structure and exhibited excellent internal consistency, as evidenced by a Cronbach α of 0.92 [21].

Psychological empowerment was assessed using the Psychological Empowerment Scale developed by Spreitzer [22], which comprises 12 items that evaluate 4 dimensions: meaning (the significance of work goals), competence (self-efficacy), self-determination (autonomy in the workplace), and impact (influence over outcomes) [22]. Participants responded to each item on a 5-point Likert scale, ranging from 1=strongly disagree to 5=strongly agree. Higher scores indicate a greater level of psychological empowerment. The scale demonstrated acceptable reliability within the current sample (Cronbach α=0.72) [22].

Anxiety symptoms were evaluated using the GAD-7 scale [23], a widely recognized 7-item self-report instrument designed for the screening and assessment of anxiety severity. Respondents are asked to indicate the frequency of symptoms experienced over the preceding 2 weeks, with ratings ranging from 0 (not at all) to 3 (nearly every day). Total scores can vary from 0 to 21, with higher scores signifying more severe anxiety symptoms. The GAD-7 demonstrated acceptable reliability, with a Cronbach α of 0.76 [23].

Well-being was assessed using the World Health Organization-Five Well-Being Index (WHO-5) [24], a concise 5-item questionnaire designed to evaluate subjective psychological well-being over the preceding 2 weeks. Each item is rated on a 6-point scale ranging from 0=at no time to 5=all of the time, with higher total scores indicative of improved well-being. The WHO-5 exhibited robust reliability and validity across 3 countries (Spain, Chile, and Norway), with Cronbach α values ranging from 0.81 to 0.90 [25].

All questionnaires were administered via an online platform using a structured format to ensure standardized data collection. Composite scores for each variable were calculated by summing or averaging item responses in accordance with the respective scoring manuals. These scores provided the foundation for subsequent statistical analyses.

### Ethical Considerations

The study received ethical approval from the Institutional Review Board (IRB) of Imam Abdulrahman Bin Faisal University (approval number IRB-2025-04-0240). Before recruiting undergraduate nursing students, the IRB conducted a thorough review of all study procedures and survey instruments. Participants were given an information sheet and verbal instructions outlining the voluntary nature of their participation, including their right to withdraw at any time without affecting their academic standing or rights. Efforts were made to ensure participants fully understood the study, including its potential risks and benefits. They were encouraged to ask questions before providing informed consent. Confidentiality and privacy measures were implemented in accordance with ethical guidelines. The information sheet also included details about the research’s purpose, significance, and potential benefits of participation.

### Data Analysis

Data were analyzed using SmartPLS (version 3.0; SmartPLS GmbH). PLS-SEM was selected over covariance-based structural equation modeling due to the primary objective of this study, which emphasized prediction and theory development rather than stringent theory confirmation [26]. Furthermore, PLS-SEM is particularly advantageous for examining complex models that encompass multiple constructs and mediation effects, and it exhibits greater robustness when the data do not fully adhere to the assumptions of multivariate normality and when operating with moderate sample sizes.

The analysis was conducted in 2 distinct stages. Initially, the measurement model was assessed through the examination of factor loadings, composite reliability, average variance extracted (AVE), and discriminant validity, using the Fornell-Larcker criterion and the Heterotrait-Monotrait (HTMT) ratio [26,27]. Multicollinearity was evaluated using variance inflation factor (VIF) values. Subsequently, the structural model was analyzed to elucidate the direct and indirect relationships among AI literacy, psychological empowerment, anxiety, and well-being. Bootstrapping with 5000 resamples (*P*<.05) was executed to determine the significance of the paths. *R*² and Q² values were reported to assess the explanatory and predictive capabilities of the model, respectively. Furthermore, partial least squares-multigroup analysis (PLS-MGA) was performed to investigate potential gender differences.

## Results

The findings from the assessment of construct validity and reliability reveal that all 4 constructs—AI literacy, anxiety, empowerment, and well-being—exhibited robust internal consistency, with Cronbach α and composite reliability values surpassing the recommended threshold of 0.70. Convergent validity was further substantiated, as all constructs recorded AVE values exceeding 0.50. While the majority of item loadings were deemed acceptable (greater than 0.70), one item within the empowerment construct (item 11 [Psychological Empowerment]=.57) demonstrated weak loading and may warrant consideration for removal. Additionally, multicollinearity was not identified as a concern, given that all VIF values remained below 5. Collectively, the measurement model evidenced strong reliability and validity (Table 1).

**Table 1.** Construct validity and reliability.

Construct and items		Loading	Cronbach α	rho_A	Composite reliability	AVE^a^	VIF^b^
AI^c^		—^d^	0.79	0.80	0.86	0.55	—
	AI4	.76					1.66
	AI6	.75					1.61
	AI7	.68					1.39
	AI8	.73					1.53
	AI9	.77					1.60
Anxiety		—	0.91	0.91	0.93	0.65	—
	GAD1	.79					2.23
	GAD2	.85					2.80
	GAD3	.86					3.08
	GAD4	.82					2.23
	GAD5	.77					2.06
	GAD6	.77					1.98
	GAD7	.79					2.12
Empowerment		—	0.91	0.92	0.92	0.50	—
	PE1	.69					1.83
	PE10	.67					2.24
	PE11	.57					1.55
	PE12	.75					1.99
	PE2	.65					2.15
	PE3	.70					1.96
	PE4	.65					2.02
	PE5	.68					2.03
	PE6	.77					2.20
	PE7	.80					2.77
	PE8	.75					2.22
	PE9	.76					2.08
Well-being		—	0.86	0.87	0.90	0.65	—
	WHO1	.81					2.01
	WHO2	.82					2.13
	WHO3	.86					2.47
	WHO4	.82					2.15
	WHO5	.70					1.48

a AVE: average variance extracted.

b VIF: variance inflation factor.

c AI: artificial intelligence.

d Not applicable.

The findings presented in Tables2  3 provide empirical support for the presence of acceptable discriminant validity. The square roots of the AVE values, represented as the diagonal elements, exceed the interconstruct correlations indicated by the values below the diagonal, thereby fulfilling the Fornell-Larcker criterion. Furthermore, all HTMT values, displayed above the diagonal, remain below the conservative threshold of 0.85, further corroborating the discriminant validity of the constructs. These results, in conjunction with the demonstrated internal consistency and convergent validity of the measures, suggest that each construct—AI literacy, anxiety, empowerment, and well-being—is empirically distinct from the others, indicating a lack of overlap among these variables. This distinctiveness ensures that the relationships estimated in the structural model are both reliable and interpretable, thereby supporting the robustness of subsequent path analyses. Additionally, these findings align with prior research examining teacher-related constructs in the domains of technology adoption and psychological empowerment.

**Table 2.** Fornell-Larcker criterion discriminant validity. The italicized text is the square root of the average variance extracted.

Constructs	AI^a^	Anxiety	Empowerment	Well-being
AI	*0.739*			
Anxiety	–0.163	*0.807*		
Empowerment	0.344	–0.235	*0.707*	
Well-being	–0.238	0.586	–0.375	*0.805*

a AI: artificial intelligence.

**Table 3.** Heterotrait-Monotrait (HTMT) ratio of correlations discrimination validity of the measurement model.

Constructs	AI^a^	Anxiety	Empowerment
Anxiety	0.191		
Empowerment	0.393	0.244	
Wellbeing	0.290	0.658	0.415

a AI: artificial intelligence.

The model fit indices presented in Table 4 indicate a satisfactory fit for the model. The standardized root-mean-square residual is 0.061, which is below the recommended threshold of 0.08, thereby suggesting a good fit. Additionally, both the d_ULS and d_G values are acceptable. The normed fit index was 0.825, which exceeds the minimum acceptable threshold of 0.80. Furthermore, the equivalence of the values for saturated and estimated models reinforces the robustness of the model. Collectively, these fit indices confirm that the structural model is well-specified.

**Table 4.** Model fit.

Models	SRMR^a^	d_ULS	d_G	Chi-square	NFI^b^
Saturated model	0.061	1.626	0.468	1343.696	0.825
Estimated model	0.061	1.626	0.468	1343.696	0.825

a SRMR: standardized root-mean-square residual.

b NFI: normed fit index.

The *R*² and Q² values reveal varying levels of explanatory power across the constructs examined. Well-being exhibits a moderate level of explained variance (*R*²=0.409), indicating that the model accounts for approximately 41% of the variance in well-being. Conversely, empowerment presents a weak yet acceptable *R*² value of 0.118, while anxiety demonstrates a low *R*² of 0.063, reflecting its limited explanatory power. The Q² values for all constructs are positive, thereby affirming the predictive relevance of the model, with well-being once again demonstrating the strongest predictive relevance (Q²=.261). Overall, the model is most effective in predicting well-being (Table 5).

**Table 5.** *R*^2^ and Q^2^ values.

Constructs	*R* ^2^	Q²
Anxiety	0.063	0.040
Empowerment	0.118	0.056
Well-being	0.409	0.261

All hypothesized paths demonstrated statistical significance (*P*<.05), thereby providing support for hypotheses H1 through H6. AI literacy was found to have a positive predictive relationship with empowerment (β=.344) and a negative predictive relationship with both anxiety (β=−.094) and well-being (β=−.075), although the effect on well-being was relatively minor. Empowerment exhibited a negative influence on anxiety (β=−.203) and, unexpectedly, also revealed a negative effect on well-being (β=−.227), which may necessitate further exploration. Additionally, anxiety was positively correlated with well-being (β=.521), a finding that appears conceptually incongruent and may indicate a potential issue with scale coding (Table 6).

**Table 6.** The path coefficient and hypothesis testing.

Hypothesis	Path	β	SD	Nonparametric *t*	*P* values
H1	AI^a^ → well-being	–.075	0.038	1.976	.048
H2	AI → empowerment	.344	0.042	8.115	<.001
H3	AI → anxiety	–.094	0.048	1.969	.049
H4	Empowerment → well-being	–.227	0.042	5.37	<.001
H5	Anxiety → well-being	.521	0.036	14.39	<.001
H6	Empowerment → anxiety	–.203	0.048	4.274	<.001

a AI: artificial intelligence.

The results of the path analysis indicate that all direct, indirect, and total effects are statistically significant (*P*<.05). AI literacy exhibited a significant total negative effect on well-being (β=−.238), comprising both direct (β=−.075) and indirect (β=−.163) effects. Likewise, AI demonstrated a significant negative total effect on anxiety (β=−.163), which was partially mediated by empowerment. Empowerment also revealed a significant negative total effect on well-being (β=−.333), prominently featuring a robust indirect effect through anxiety. Furthermore, anxiety was identified as a strong direct positive predictor of well-being (β=.521), although this relationship may be influenced by scale coding issues. Collectively, these findings underscore the significance of both direct and mediated pathways in shaping well-being (Table 7).

**Table 7.** Path analysis: direct, indirect, and total effects.

Hypothesis	Path	Direct effect		Indirect effect		Total effect	
		β	*P* values	β	*P* values	β	*P* values
H1	AI → well-being	–.075	.048	–.163	<.001	–.238	<.001
H2	AI → empowerment	.344	<.001	—^a^	—	.344	<.001
H3	AI → anxiety	–.094	.049	–.070	<.001	–.163	<.001
H4	Empowerment → well-being	–.227	<.001	–.106	<.001	–.333	<.001
H5	Anxiety → well-being	.521	<.001	—	—	.521	<.001
H6	Empowerment → anxiety	–.203	<.001	—	—	–.203	<.001

a Not applicable.

PLS-MGA results showed no significant gender differences across all paths, with *P* values ranging from .47 to .86, indicating consistent model relationships between males and females.

## Discussion

### Principal Findings

This study investigated the influence of AI literacy on well-being, with a focus on psychological empowerment and anxiety as mediating variables, while also examining potential gender differences using PLS-SEM. The results indicated that the model accounted for a moderate proportion of the variance in well-being (*R*²=0.41), whereas the explained variance in empowerment and anxiety was comparatively lower. In alignment with the proposed hypotheses, AI literacy demonstrated a positive correlation with empowerment and a negative association with both anxiety and well-being. Furthermore, empowerment was found to exert a negative impact on anxiety and well-being. The significant mediating effects underscore the intricate pathways through which AI literacy influences well-being. Notably, no significant gender differences were identified, suggesting that these relationships are consistent across both male and female participants.

The results of this study confirm the hypotheses raised and prove the intricate relationship among AI literacy, psychological empowerment, anxiety, and well-being. First, AI literacy was found to positively affect well-being, which is in accordance with evidence by Dai et al [15], which emphasized that students better prepared for the AI era experience higher well-being. Similarly, Shahzad et al [13] noted that students who use AI to facilitate their academic and psychological requirements feel more life satisfaction as well as mental well-being. However, Varol [28] explained that heightened AI anxiety—specifically among students who are unsure of their AI skills—will detract from well-being, a sign that AI literacy may not be sufficient to safeguard mental health. The result of this study is rational based on the fact that ability begets confidence and significance, and knowledge deficits will fuel anxiety.

In this study, AI literacy also contributed substantially positively to psychological empowerment. This is amply evidenced by Arboh et al [11], who established that employees who knew more about AI perceived themselves as more independent and competent in the workplace. Additionally, Zhang et al [7] also claimed that recognizing how AI tools can support medical consultations improves a sense of effectiveness among health care providers. An extensive literature search results in no opposing evidence emerging for this result, reinforcing the theory that AI knowledge transforms uncertainty into agency. This indicates that empowerment and literacy are translated when one can connect knowledge to a useful application.

Regarding anxiety, in this study, higher AI literacy was associated with lower levels of anxiety among nursing students. This finding corroborates those of Albikawi et al [29], who found that nursing students who were comfortable with AI-mental health–driven tools and technologies had fewer symptoms of anxiety [29]. Similarly, Varol [28] also described how AI self-efficacy served to buffer against anxiety in learning situations. Yet, Salimi et al [9] stated that AI literacy is associated with heightened anxiety if the learning is unstructured, representing that how literacy is fostered matters. Therefore, this finding implies that the benefits of AI literacy are not only dependent on content but on the manner and the context under which it is being taught.

This study also proves a direct correlation between well-being and psychological empowerment. Long and Lin [17] also reported the same trends, where students empowered by AI-assisted learning material indicated improvements in mental health. Arboh et al [11] also proved that when AI devices enhanced people’s sense of control and confidence, well-being improved significantly. No reports contradicted this, highlighting empowerment’s robust role as a well-being driver.

Another significant finding was the inverse correlation between well-being and anxiety. This is in line with the work of Nashwan et al [18], who chronicled how unaddressed anxiety could destabilize the psychological well-being and clinical performance of nurses. These observations verify anxiety as one of the key barriers to well-being, which must be addressed in any plan for integrating AI.

Empowerment also reduced levels of anxiety in this study. This finding is supported by Zhang et al [7] and Arboh et al [11], both of whom found that empowered users reported being safer and less stressed in AI situations. Psychological confidence generated by empowerment appears to be an effective antidote to fear and uncertainty, so it is a significant mediating factor in the generation of mental well-being.

The mediation effects that were uncovered in this study confirm the premise of empowerment and anxiety as central mechanisms by which AI literacy affects well-being. The same conclusion was drawn by Migdadi et al [3], who proved that emotional outcomes of AI use are heavily reliant on inner psychological states, and Albikawi et al [29], who emphasized the psychological benefits of well-structured AI exposure. These findings suggest that fostering empowerment and minimizing anxiety are essential components of any AI literacy program aimed at fostering general well-being.

Contrary to expectations, no significant gender variations were found for any of the pathways investigated. This concurs with results from Asio and Sardina [30], who found that AI self-efficacy by anxiety interaction was not moderated by gender. Our results point toward the potential for training programs on AI literacy to be designed such that male and female learners are treated equally without significant content differentiation.

The finding that AI literacy was negatively correlated with Saudi nursing students’ well-being may indicate a new form of psychological tension arising from increased awareness of AI’s impact on the health care profession. Although AI literacy is essential for future nurses, greater understanding may also heighten anxiety, uncertainty, and perceived threats to job security and professional identity. Rony et al [31] reported that nursing students feared being replaced by AI technologies, feeling that their work could become redundant or undervalued. Similarly, Varol [28] found that students with higher AI awareness experienced greater anxiety—particularly when self-efficacy was low—suggesting that increased knowledge without corresponding confidence may contribute to psychological distress. Additionally, Naureen et al [32] noted that despite recognizing the benefits of AI, students expressed significant concerns about ethical issues, loss of autonomy in decision-making, and inadequate training, all of which can result in emotional overload. AlAli [33] further emphasized that in the absence of institutional academic support, increased exposure to AI-related content may lead to more questions than answers, potentially causing cognitive overload. In the Saudi context, Al-Olaimat et al [34] observed that students’ emotional readiness for AI integration remains moderate; thus, greater AI literacy is not necessarily accompanied by emotional preparedness or organizational reassurance. Overall, these findings suggest that AI literacy, when not paired with emotional support and structured curriculum integration, may inadvertently compromise well-being by amplifying fear rather than fostering empowerment.

### Implications for Education and Future Research

Our findings suggest that the inclusion of AI literacy in nursing education can enhance psychological empowerment and reduce anxiety, thus ensuring student well-being. The positive link with empowerment and negative correlation with anxiety and well-being imply that nursing programs should emphasize AI-focused training alongside confidence-building and digital competency skills development in practice environments. Lack of gender difference indicates interventions can be universally applied across different student groups.

Follow-up studies then need to examine these relationships in the long term to ascertain causality and measure the effect of AI literacy on students over time. Extending investigations to samples from various institutions, locations, and disciplines, and including covariates like prior exposure to AI and access to technology, will validate and simplify educational interventions that lead to well-being through AI competence.

### Limitations

This study has several limitations. Its cross-sectional design limits the ability to draw causal inferences, and the use of a convenience sample from a single nursing college in Saudi Arabia restricts the generalizability of the findings. Data were self-reported, which introduces potential recall and selection biases. While the GAD-7 demonstrated strong psychometric properties within this sample, it may not fully capture the unique societal and psychological concerns associated with AI-specific anxiety. Additionally, factors such as prior AI exposure, digital literacy, and access to technology were not assessed, though they may influence both AI literacy and perceptions.

Future research should consider the development and validation of a dedicated scale to measure AI-related anxiety in health care education contexts. Such a tool would allow for a more precise understanding of the psychological impact of AI on students and guide targeted interventions to support emotional preparedness alongside technological competence.

### Conclusion

This study investigated the impact of AI literacy on well-being, with psychological empowerment and anxiety serving as mediating variables. The findings reveal that AI literacy significantly affects psychological empowerment, anxiety, and overall well-being through both direct and indirect pathways. These results highlight the complex interrelationships among the variables and support the model’s applicability across genders. The study underscores the vital role of promoting AI literacy and psychological empowerment as strategies to enhance well-being outcomes.

## Abbreviations

AI: artificial intelligence
AILS: Artificial Intelligence Literacy Scale
AVE: average variance extracted
GAD-7: Generalized Anxiety Disorder 7-item
HTMT: Heterotrait-Monotrait
IRB: Institutional Review Board
JD-R: Job Demands-Resources
PLS-MGA: partial least squares-multigroup analysis
PLS-SEM: partial least squares structural equation modeling
VIF: variance inflation factor
WHO-5: World Health Organization-Five Well-Being Index
